# Construction of a right ventricular function assessment model in patients undergoing invasive mechanical ventilation based on VExUS grading and the classification and regression tree algorithm

**DOI:** 10.3389/fcvm.2025.1608210

**Published:** 2025-09-04

**Authors:** Jun Gao, Pan Gao, Yuanyuan Du, Jianjun Zhang, Jinrong Wang

**Affiliations:** ^1^ Department of Critical Care Medicine, Harrison International Peace Hospital, Hengshui, China; ^2^ Graduate College, Hebei Medical University, Shijiazhuang, China

**Keywords:** invasive mechanical ventilation, right ventricular function, venous excess ultrasound (VExUS) grading system, classification and regression tree (CART) algorithm, model

## Abstract

**Objective:**

Investigate the correlation between right ventricular function ultrasound indicators and the Venous Excess Ultrasound (VExUS) grading system in patients undergoing invasive mechanical ventilation (IMV), and develop a right ventricular function assessment model using VExUS grading and the Classification and Regression Tree (CART) algorithm.

**Methods:**

This cross-sectional study collected data from patients in the Department of Critical Care at Harrison International Peace Hospital who underwent tracheal intubation within 24–48 h from October 2023 to December 2024. Collected information comprised general clinical data, hematological indices, hemodynamic parameters, severity scores, right ventricular function ultrasound indicators, the maximum diameter of the inferior vena cava (IVCdmax), and Doppler spectra from the hepatic, portal and intrarenal veins. Patients were divided into two groups (VExUS 0 and VExUS ≥1) based on VExUS grading system to reflect the presence or absence of venous congestion. Baseline data were compared between groups, and Spearman's correlation analysis was performed to assess the relationship between VExUS grading and right ventricular function ultrasound indicators. Subsequently, a right ventricular function assessment model was constructed using the CART algorithm.

**Results:**

A total of 80 patients were enrolled, with 26 in the VExUS 0 group and 54 in the VExUS ≥1 group. In the VExUS ≥1 group, the proportion of male patients, levels of NT-proBNP, cTnI, central venous pressure, cumulative fluid balance, and the usage rate of diuretics were significantly higher than in the VExUS 0 group (*P* < 0.05). IVCdmax was positively correlated with VExUS grading (*r* = 0.773, *P* < 0.05), whereas tricuspid annular plane systolic excursion (TAPSE) and the tricuspid E-wave peak were negatively correlated (*r* = −0.670 and −0.648, respectively; *P* < 0.05). In the CART model analysis, the importance of the variables was ranked as: IVCdmax > TAPSE > tricuspid E-wave peak. When validated with a test set, the model achieved an overall accuracy of 87.5%.

**Conclusion:**

In patients undergoing IMV, IVCdmax, TAPSE, and the tricuspid E-wave peak show strong correlations with VExUS grading. A right ventricular function assessment model based on VExUS grading and the CART algorithm can effectively evaluate right ventricular performance and may serve as a useful tool in identifying venous congestion.

## 1 Introduction

Invasive mechanical ventilation (IMV) serves as a crucial supportive measure in the treatment of critically ill patients, effectively maintaining respiratory function; however, its application has significant and far-reaching effects on the circulatory system, particularly on right ventricular function (1). The right ventricle (RV), as a key link between the systemic and pulmonary circulations, directly influences patient oxygenation and hemodynamic stability; therefore, accurate assessment of right ventricular function is essential for optimizing ventilation strategies and improving patient outcomes.

In recent years, the widespread adoption of critical care ultrasound has enriched the methods available for assessing right ventricular function. Echocardiography provides dynamic and intuitive information on right ventricular structure and function—such as right ventricular fractional area change and tricuspid annular plane systolic excursion (TAPSE)—and has become an important clinical tool for evaluating right ventricular performance (2). However, these indicators in critically ill patients may be affected by factors such as mechanical ventilation, challenges in image acquisition, and operator experience. Therefore, identifying a simple, quantifiable method that correlates well with ultrasound evaluation parameters for right ventricular function is an important focus of current clinical research.

Recently, the introduction of bedside ultrasound techniques—particularly the Venous Excess Ultrasound (VExUS) grading system has provided a novel approach for assessing right ventricular function (3–5). The VExUS grading system integrates the diameter of the inferior vena cava (IVC) with abnormal Doppler waveforms of the hepatic, portal, and intrarenal veins to classify the degree of venous congestion into grades 0–3; higher grades indicate more severe congestion and quantitatively reflect the downstream effects of elevated right atrial pressure (RAP) (6). Persistent venous congestion has been associated with impaired organ perfusion, increased risk of acute kidney injury and worse outcomes in critically ill patients (7, 8). Clinical studies have shown that signs of venous congestion are associated with longer ICU stays, increased need for renal replacement therapy and higher mortality rates (9, 10). Therefore, early identification and mitigation of venous congestion are essential to prevent further hemodynamic compromise and organ dysfunction.

Given the close relationship between venous congestion and right ventricular function, the VExUS grading system may serve as an important tool for assessing right ventricular dysfunction; however, its applicability in IMV patients and its correlation with right ventricular function indicators remain unclear. To address this issue, the present study explores the correlation between right ventricular function ultrasound indicators and VExUS grading in IMV patients, and attempts to develop a right ventricular function assessment model based on VExUS grading and the Classification and Regression Tree (CART) algorithm which can provide a basis for clinical stratification and management.

## 2 Materials and methods

### 2.1 Study subjects

**Figure 1:**
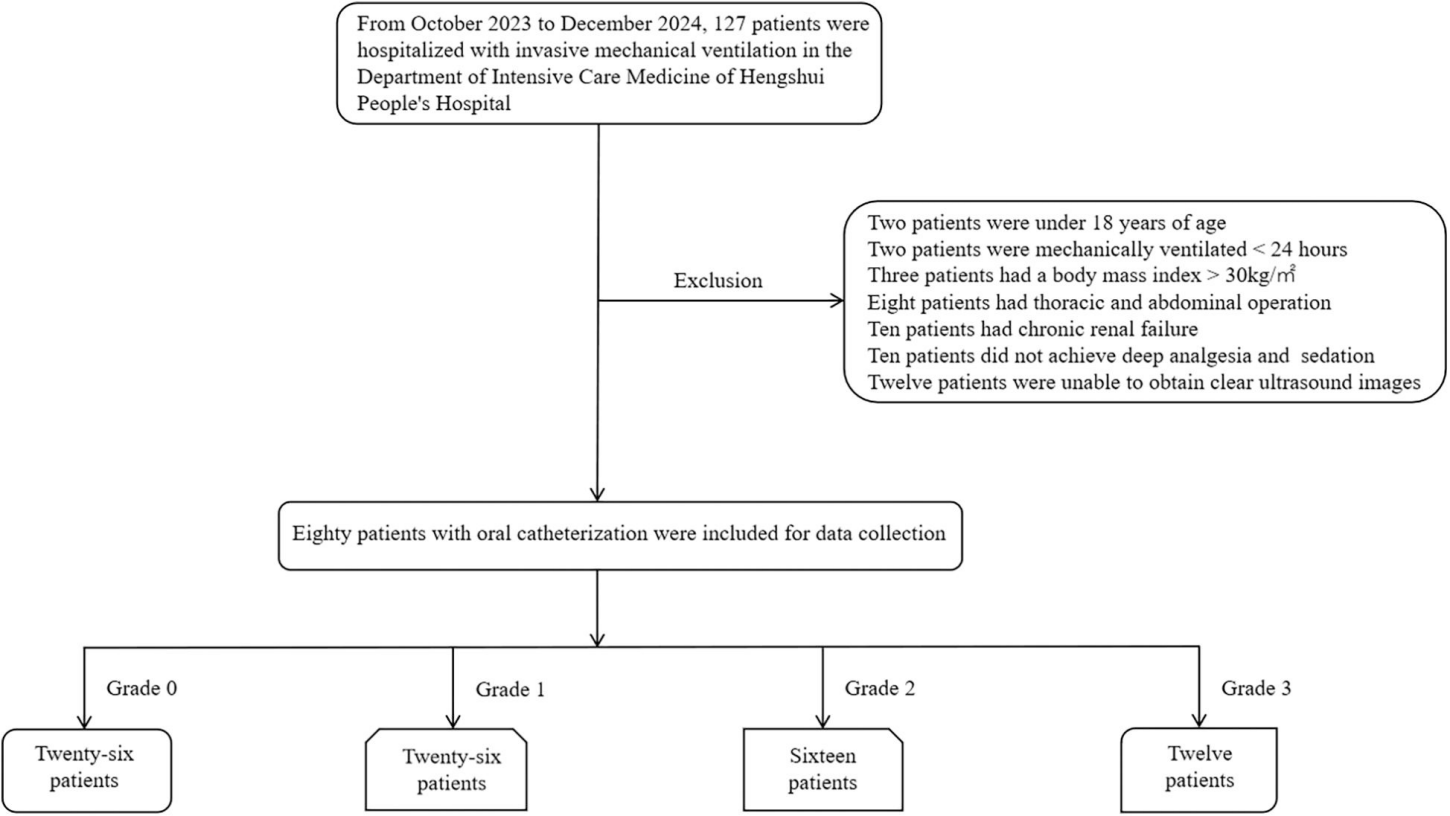
Enrollment and exclusion flowchart.

In this cross-sectional observational study, 127 patients who received IMV treatment and underwent ultrasound examinations of the heart, inferior vena cava, hepatic, portal, and intrarenal veins in the Department of Critical Care at Harrison International Peace Hospital from October 2023 to December 2024 were initially enrolled; 47 cases were excluded, resulting in a final sample of 80 patients. Based on the presence of venous congestion as determined by VExUS grading, the patients were divided into two groups: the VExUS 0 group (26 patients) and the VExUS ≥1 group (54 patients), as shown in Figure 1.

**Inclusion criteria:** age between 18 and 90 years; oral tracheal intubation with mechanical ventilation for 24 to 48 h; deep sedation and analgesia, resulting in no spontaneous breathing.

**Exclusion criteria:** Chest wall deformities; a body mass index >30 kg/m^2^; a history of thoracic or abdominal surgery; pleural or peritoneal effusions or large tumors; severe hepatic, renal, or other organ failure, or end-stage malignancy; right ventricular outflow tract or pulmonary valve stenosis, outflow tract obstruction, ventricular septal defect, or patent ductus arteriosus; unclear or incomplete images; and non-standard or abnormal data measurements. These exclusion criteria were applied to minimize confounding factors and to ensure adequate ultrasound image quality, such as obesity, effusions, prior surgery and advanced organ failure may interfere with Doppler signal acquisition or independently affect venous flow patterns.

### 2.2 Research methods

For the 80 enrolled patients, deep sedation and analgesia were administered to achieve a state of no spontaneous breathing while ensuring stable vital signs. Within 24–48 h after initiating mechanical ventilation via oral tracheal intubation, data were collected on general clinical indicators [age, gender, body mass index (BMI), body temperature, respiratory rate, duration of mechanical ventilation, total hospitalization time, and previous medical history], hematological indicators (Oxygenation Index (OI), Partial Pressure of Carbon Dioxide (PCO₂), N-terminal pro-B-type Natriuretic Peptide (NT-proBNP), Cardiac Troponin I (cTnI), Creatine Kinase-MB Isoenzyme(CK-MB)), hemodynamic parameters [heart rate, Arterial Oxygen Saturation (SO_2_), Central Venous Pressure (CVP), Mean Arterial Pressure (MAP), lactate, cumulative fluid balance, and the use of vasoactive drugs, inotropes, diuretics, CRRT, and drainage procedures], severity scores [Sequential Organ Failure Assessment (SOFA) andAcute Physiology and Chronic Health Evaluation II (APACHE II)], and right ventricular function ultrasound indicators (IVCdmax, Inferior Vena Cava Collapsibility Index (*Δ*IVC), Right Ventricular End-Diastolic Area (RVEDA), Right Atrial End-Diastolic Area (RAEDA), Right Ventricular Fractional Area Change (FAC), Tricuspid Annular Plane Systolic Excursion (TAPSE), Tricuspid Valve Early Diastolic Inflow Peak Velocity (E-wave peak), Tricuspid Valve Late Diastolic Inflow Peak Velocity (A-wave peak), Tricuspid E/A Ratio (E/A ratio), Pulmonary Artery Systolic Pressure (PASP), Eccentricity Index (EI), Right Ventricular Free Wall Thickness (RVFW)). In addition, the diameter of the inferior vena cava, Doppler spectra of the hepatic, portal, and intrarenal veins, were recorded (Supplementary Figures S1–S4). During the study, each patient's ultrasound parameters were measured three times by the same sonographer within a single time period, and the average value was used for analysis.

### 2.3 Statistical methods

Data were analyzed using SPSS 27.0. Continuous variables were tested for normality using the Shapiro–Wilk test; those with a normal distribution were expressed as mean ± standard deviation (SD) and compared using the independent samples t-test, while non-normally distributed data were expressed as medians [M (P25, P75)] and compared using the Wilcoxon Mann–Whitney rank-sum test. Categorical data were expressed as counts (percentages) and compared using the chi-square test, chi-square correction, or Fisher's exact test. The correlation between VExUS grading and right ventricular function ultrasound indicators was assessed using Spearman's correlation test. Data analysis was further performed using MATLAB R2024b, and the 80 patients were randomly divided into a training set (*n* = 64) and a test set (*n* = 16) in an 8:2 ratio. A CART algorithm was employed for the analysis, selecting the feature with the highest information gain as the classification attribute and recursively expanding the branches of the decision tree to construct the model. To evaluate the overall classification performance of the model, performance metrics including accuracy, precision, recall, and F1 score were used, and a confusion matrix and ROC curve were plotted using the test set for further validation; a *P*-value < 0.05 was considered statistically significant.

## 3 Results

### 3.1 Comparison of baseline data

In the VExUS ≥1 group, the proportion of male patients and levels of NT-proBNP, cTnI, CVP, cumulative fluid balance, as well as the rate of diuretic usage were significantly higher than those in the VExUS 0 group (*P* < 0.05; see Table 1).

**Table 1:** Comparison of baseline data between the VExUS 0 group and the VExUS ≥1 group.

Variable	VExUS grading		Test statistic	*P* value
	Grade 0 (*n* = 26)	Grade ≥1 (*n* = 54)		
General clinical indicators				
Age (years)	73.00 (69.00, 77.50)	72.00 (59.00, 77.00)	0.864	0.387
Male	10 (38.50)	42 (77. 80)	11.924	<0.001
BMI (kg/m²)	25.25 (22.95, 29.32)	24.89 (22.20, 27.68)	0.946	0.344
Body Temperature (°C)	37.22 ± 0.85	36.93 ± 0.69	1.631	0.107
Respiratory Rate (breaths/min)	18.00 (15.00, 20.50)	18.00 (15.00, 24.00)	0.189	0.850
Duration of Mechanical Ventilation (days)	11.00 (4.00, 12.50)	9.00 (6.00, 11.00)	1.364	0.173
Total Hospitalization Time (days)	22.00 (9.75, 38.00)	14.00 (7.00, 22.00)	1.461	0.144
Hypertension	20 (76.90)	34 (63.00)	1.559	0.212
Diabetes	8 (30.80)	18 (33.30)	0.053	0.819
Coronary Heart Disease	8 (30.80)	24 (44.40)	1.368	0.242
Hematological indicators				
OI (mmHg)	258.06 ± 62.42	243.64 ± 93.90	0.815	0.418
PCO_2_ (mmHg)	33.90 (28.18, 41.00)	37.40 (32.10, 42.10)	−0.884	0.377
NT-proBNP (ng/L)	1301.00 (352.25, 6433.50)	4386.00 (831.00, 25000.00)	2.104	0.035
cTnI (ng/ml)	30.00 (3.75, 120.50)	474.00 (42.00, 3839.90)	4.380	<0.001
CK-MB (ng/ml)	9.00 (7.75, 15.25)	14.00 (7.00, 29.00)	1.297	0.195
Hemodynamic indicators				
Heart Rate (beats/min)	88.00 (69.75, 105.75)	89.00 (71.00, 100.00)	0.062	0.951
SO_2_ (%)	97.20 (96.60, 98.18)	97.10 (96.30, 98.30)	0.041	0.967
CVP (cmH_2_0）	5.00 (4.00, 5.25)	7.00 (6.00, 9.00)	5.301	<0.001
MAP (cmH_2_0)	93.33 (79.08, 108.17)	84.33 (76.33, 104.67)	0.534	0.593
Lac (mmol/L)	1.47 (1.19, 2.24)	1.78 (1.15, 2.38)	0.822	0.411
Cumulative Fluid Balance (ml)	651.21 ± 818.18	−135.19 ± 1263.91	3.343	0.001
Use of Vasoactive Drugs	14 (53.80)	30 (55.60)	0.021	0.886
Use of Inotropic Agents	0 (0.00)	8 (14.80)	2.792	0.095
Use of Diuretics	14 (53.80)	16 (29.60)	4.391	0.036
Use of CRRT	8 (30.80)	18 (33.30)	0.053	0.819
Use of Percutaneous Drainage	0 (0.00)	6 (11.11)	1.727	0.189
Severity indices				
SOFA Score (points)	5.00 (2.00, 7.25)	6.00 (4.00, 9.00)	1.448	0.148
APACHE II Score (points)	17.00 (14.75, 26.25)	23.00(16.00, 30.00)	1.709	0.087

Continuous variables that follow a normal distribution are expressed as Mean ± SD (test statistic: *t*); those not normally distributed are expressed as M (P25, P75) (test statistic: z); categorical data are expressed as *N* (%) (test statistic: *χ*²).

### 3.2 Analysis of the correlation between VExUS grading and right ventricular function ultrasound indicators

IVCdmax was positively correlated with VExUS grading (*r* = 0.773, *P* < 0.05), whereas TAPSE and the tricuspid E-wave peak were negatively correlated with VExUS grading (*r* = –0.670 and *r* = –0.648, respectively; *P* < 0.05; see Table 2).

**Table 2:** Correlation analysis between VExUS grading and right ventricular function indicators.

Variables	VExUS grading	
	r value	*P* value
IVCdmax	0.773	<0.001
ΔIVC	−0.108	0.342
RVEDA	0.136	0.230
RAEDA	0.072	0.527
FAC	−0.013	0.909
TAPSE	−0.670	<0.001
Tricuspid E-wave	−0.648	<0.001
Tricuspid A-wave	−0.188	0.096
Tricuspid E/A ratio	−0.157	0.165
PASP	0.061	0.593
EI	−0.004	0.973
RVFW	0.031	0.785

### 3.3 Construction of the right ventricular function assessment model

**Figure 2:**
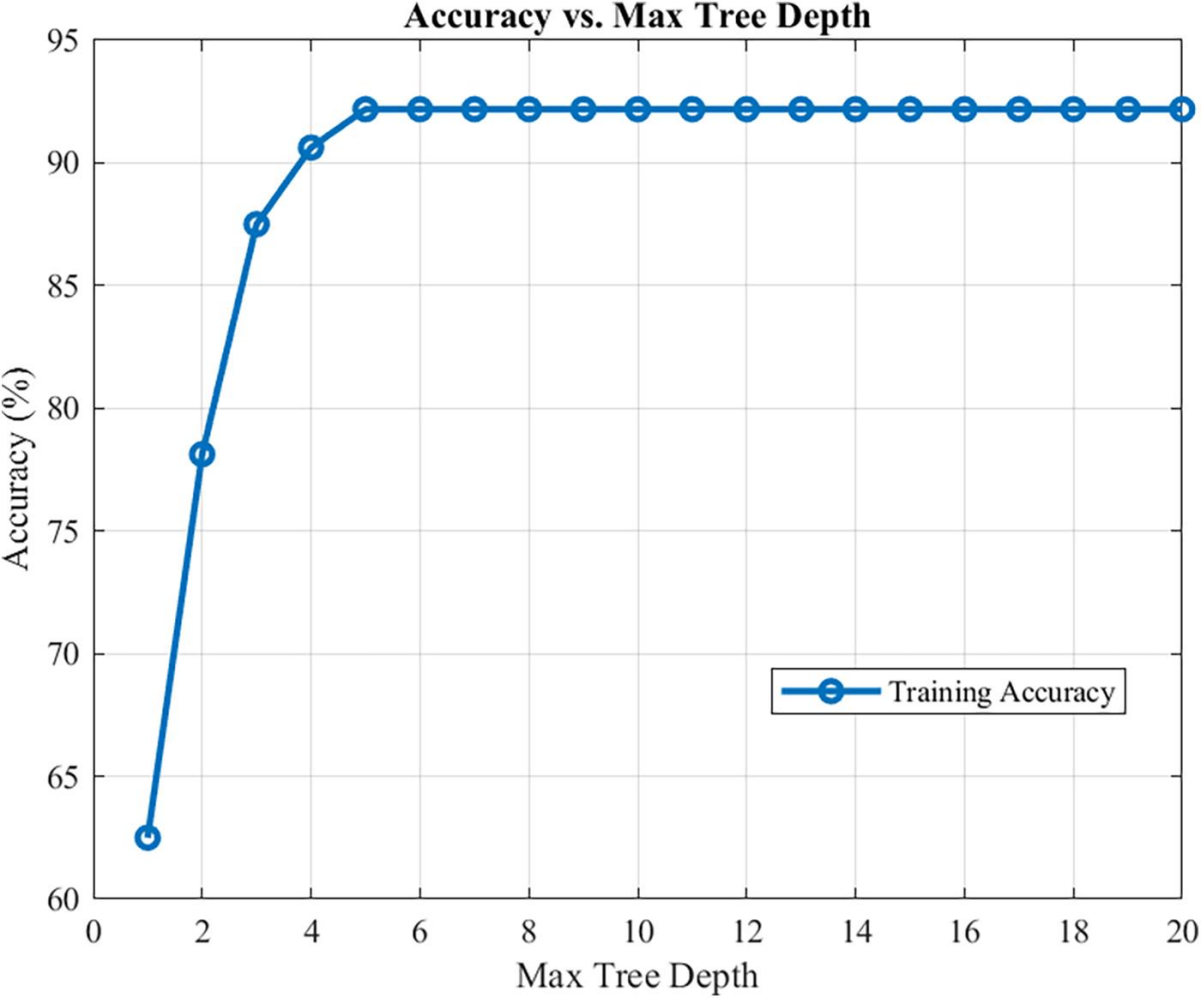
Comparison of accuracy at different CART model depths.

**Figure 3:**
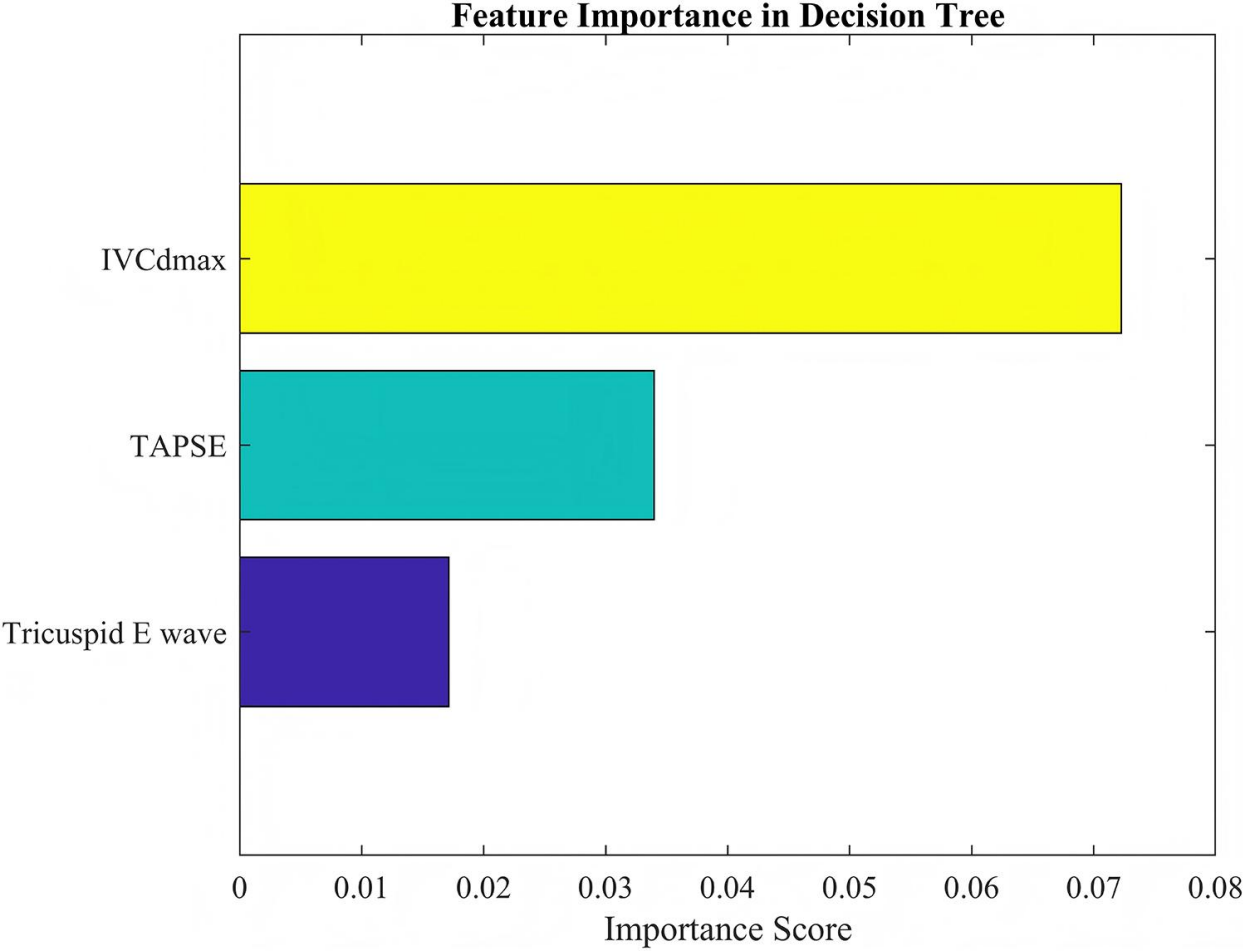
Feature importance.

**Figure 4:**
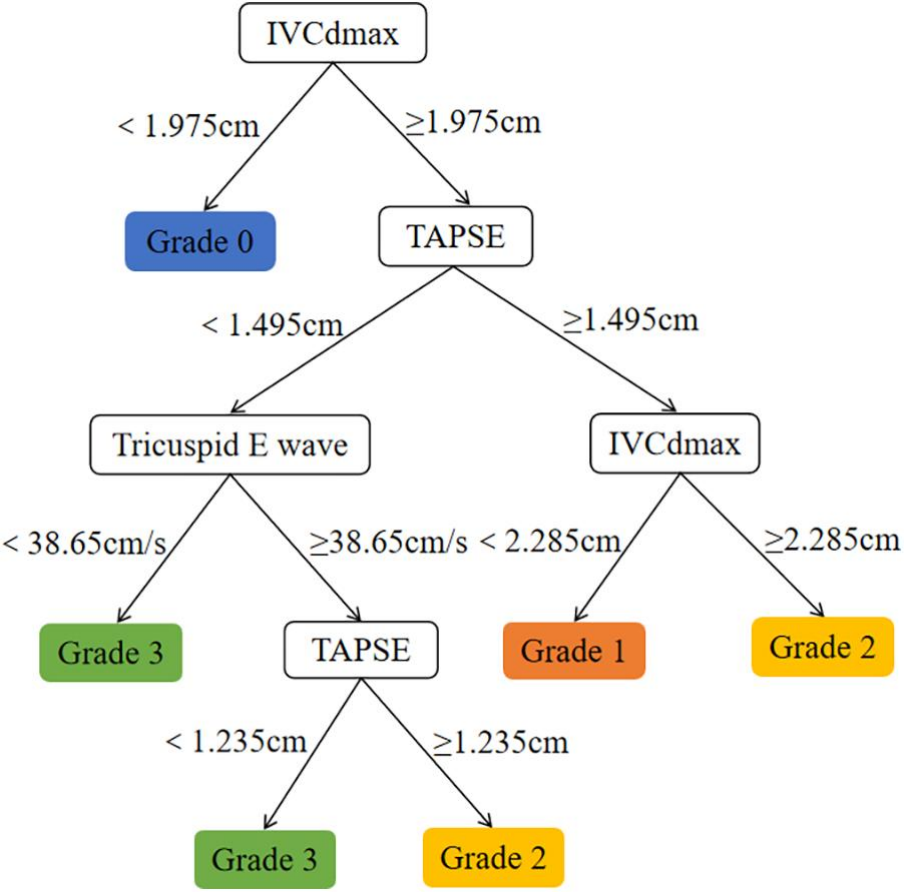
CART model.

**Figure 5:**
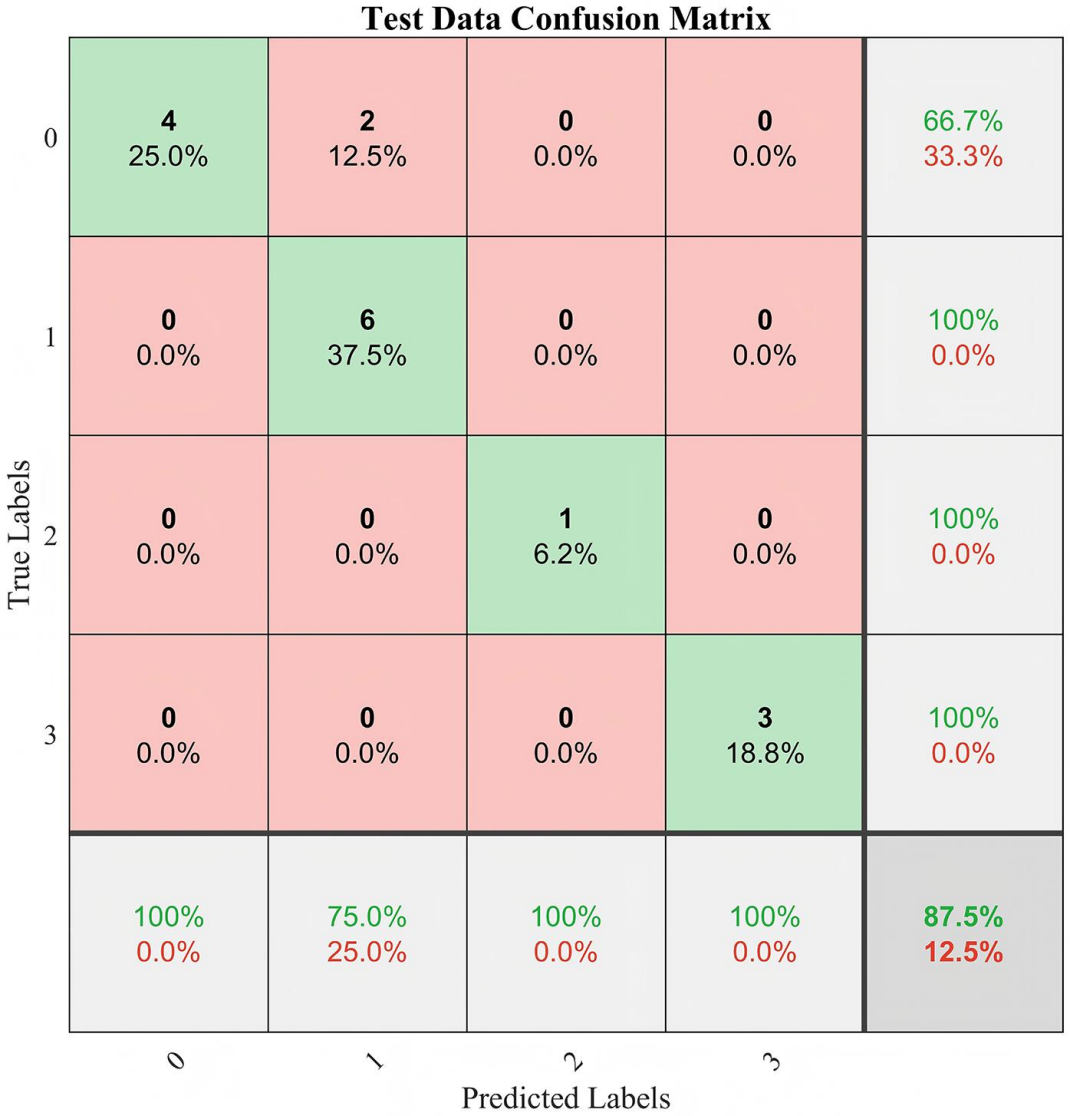
Confusion matrix.

**Figure 6:**
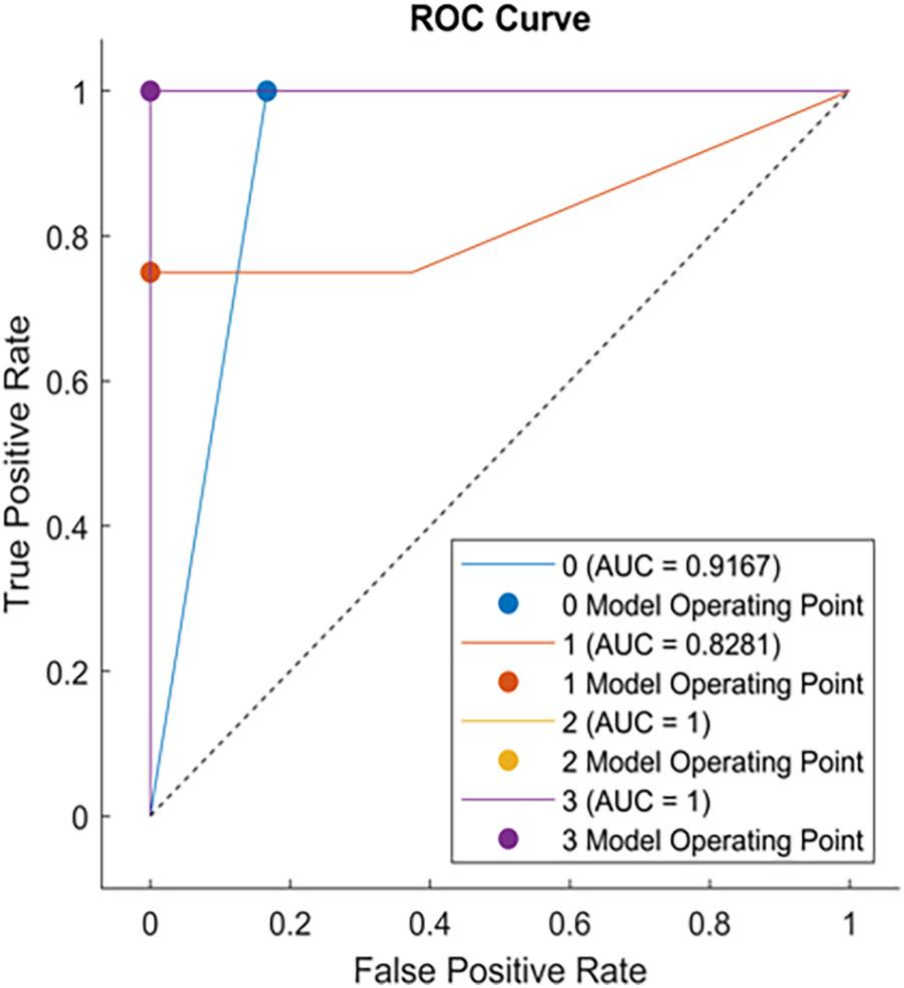
ROC curve.

Comparison of CART models at different depths revealed that a tree depth of ≥5 yielded the highest accuracy; therefore, a 5-level CART model was constructed (Figure 2). The feature importance of the included variables was ranked as follows: IVCdmax > TAPSE > tricuspid E-wave peak (Figure 3). In the constructed CART model, the right ventricular function ultrasound indicators associated with VExUS grading were stratified, with IVCdmax determined as the root node (node value = 1.975), 6 leaf nodes, and a tree depth of 5. The leaf nodes were further divided into four groups based on VExUS grading—blue boxes for grade 0, orange boxes for grade 1, yellow boxes for grade 2, and green boxes for grade 3 (Figure 4). When applied to the test set, the model achieved an overall accuracy of 87.5%. For VExUS grade 0, the precision, recall, and F1 score were 66.7%, 100%, and 80.0%, respectively; for grade 1, they were 100%, 75.0%, and 85.7%; and for grades 2 and 3, all metrics were 100% (Table 3). Furthermore, using a confusion matrix, the classification accuracy was 87.5% with an error rate of 12.5% (Figure 5), and ROC curve analysis yielded an area under the curve (AUC) of 0.917 for the VExUS grade 0 model, 0.828 for the grade 1 model, and 1 for both the grade 2 and grade 3 models (Figure 6).

**Table 3:** Performance metrics of the model.

Group	Precision	Recall	F1 score	Sample size
Grade 0	66.7%	100%	80.0%	26
Grade 1	100%	75.0%	85.7%	26
Grade 2	100%	100%	100%	16
Grade 3	100%	100%	100%	12
Accuracy on Test Set			87.5%	

## 4 Discussion

The VExUS grading system, by assessing the congestion status of the hepatic, portal, and intrarenal veins from multiple dimensions, can sensitively reflect the retrograde transmission effect of elevated RAP on the venous system (11), thereby providing clinicians with a simple, rapid, and highly reproducible tool for evaluating right ventricular function—particularly suited for bedside monitoring of IMV patients. Our study demonstrated a significant correlation between VExUS grading and IVCdmax, TAPSE, as well as the tricuspid E-wave peak, indicating that VExUS grading can effectively quantify the status of right ventricular function.

We found that the VExUS ≥1 group had a significantly higher proportion of male patients and elevated levels of NT-proBNP, cTnI, CVP, cumulative fluid balance, as well as a higher rate of diuretic use compared to the VExUS 0 group, suggesting a direct association between the severity of venous congestion and right ventricular dysfunction. The higher proportion of males in the VExUS ≥1 group is consistent with previous studies. Melenovsky et al. (12) reported that male gender is an independent predictor of right ventricular dysfunction, possibly due to androgens promoting myocardial fibrosis or increasing pulmonary arterial pressure sensitivity. Furthermore, due to anatomical differences, male patients may have a reduced compensatory capacity for volume overload (13), which can facilitate the progression to a higher VExUS grade. Elevated levels of NT-proBNP and cTnI reflect the dual mechanisms of myocardial stress and injury, and our study found that these biomarkers are positively correlated with VExUS grading. As a marker of ventricular wall tension, elevated NT-proBNP levels suggest that the right ventricle compensates to counteract volume overload, whereas increased cTnI levels may stem from right ventricular microcirculatory ischemia or oxidative stress injury, particularly in the presence of compromised coronary perfusion (14). The significantly positive cumulative fluid balance observed in the VExUS ≥1 group indicates that fluid overload is a key driving factor for venous congestion. In patients with right ventricular dysfunction, the Frank-Starling curve tends to flatten, and excessive fluid administration may lead to right ventricular dilation and a decrease in cardiac output (15). Previous research has shown that each 1 L increase in cumulative fluid volume raises CVP by approximately 2–3 mmHg (16), further exacerbating visceral congestion, which is consistent with the higher CVP observed in the VExUS ≥1 group of our study. The higher rate of diuretic use in the VExUS ≥1 group reflects proactive clinical intervention against venous congestion. Consistent with the findings of Jury et al. (5), diuretics can alleviate hepatic congestion and peripheral edema, thereby reducing right ventricular preload and improving septal motion.

Operator-dependent variability is an inherent limitation in ultrasound-based assessments, including the VExUS grading system and the right ventricular function measurements. Differences in probe positioning, image acquisition angles, and Doppler waveform interpretation can lead to measurement discrepancies among operators (17, 18). In this study, all ultrasound examinations and Doppler measurements were performed by a single experienced sonographer to minimize inter-operator variability, and each parameter was measured three times with the average value used for analysis. But this approach limits the generalizability of our findings to settings with different operators of varying experience levels.

IVCdmax is an indirect indicator of RAP which can reflect the elevation of CVP and an increase in venous return resistance. Our study found that IVCdmax is positively correlated with VExUS grading, indicating that in mechanically ventilated patients, increased intrathoracic pressure leads to reduced right ventricular preload and increased afterload, further exacerbating venous congestion. This finding aligns with the concept proposed by Souligny et al. (4), which integrates IVC diameter with visceral venous Doppler waveforms in VExUS grading, underscoring IVC dilation as a core indicator of venous congestion. TAPSE is a key indicator for assessing right ventricular longitudinal systolic function, and a decrease in TAPSE suggests right ventricular systolic dysfunction. Our study found that TAPSE is negatively correlated with VExUS grading, indicating that impaired right ventricular systolic function leads to decreased cardiac output, which, through neurohumoral regulation, increases fluid retention and further aggravates venous congestion. Additionally, positive pressure ventilation during mechanical ventilation may further impair right ventricular ejection by increasing pulmonary vascular resistance (19). This observation echoes the findings of Bhardwaj et al. (20), which showed that higher VExUS grading is associated with an increased risk of acute kidney injury due to right ventricular dysfunction. The tricuspid E-wave peak reflects the early diastolic filling capacity of the right ventricle, and its reduction suggests diastolic dysfunction. Our study demonstrated that the tricuspid E-wave peak is negatively correlated with VExUS grading, indicating that decreased right ventricular compliance restricts diastolic filling which in turn increases venous return resistance and visceral congestion, thereby influencing VExUS grading. It is noteworthy that fluctuations in intrathoracic pressure during mechanical ventilation may further impair right ventricular diastolic function by altering trans-mural pressure (21). This mechanism is consistent with the pathophysiological basis underlying abnormal Doppler waveforms in the portal and intrarenal veins observed in VExUS grading. The IVCdmax was selected as the primary node for classifying VExUS grades in our CART model. However, all patients with VExUS grades > 0 showed IVCdmax greater than 2.1 cm, thus there was a coupling effect between IVCdmax and VExUS grade. Although a coupling effect between IVCdmax and VExUS grading may have led to an overestimation of IVCdmax's importance in the CART model, this does not diminish the value of our findings. IVCdmax remains a clinically relevant marker of venous congestion, and the model also incorporated functional indicators such as TAPSE and tricuspid E-wave peak, providing complementary information. This suggests the model reflects both structural and functional aspects of right ventricular assessment.

Many models have demonstrated excellent predictive accuracy in clinical settings, such as ensemble methods (e.g., random forests, gradient boosting machines) and deep learning methods (22–24), but their complexity often hinders clinical interpretability and clinical application. In contrast, the CART algorithm provides a transparent and intuitive decision-making process that is easy for clinicians to understand and implement (25). This interpretability is critical for clinical tools that require real-time decision making. In addition, unlike traditional regression models (26), CART is able to naturally handle nonlinear relationships and interactions without extensive data preprocessing or assumptions. Although CART may have lower predictive accuracy compared to ensemble or deep learning models, CART's balance between interpretability and performance justifies its standalone application in this study.

The CART model constructed in this study showed high discriminative ability in distinguishing the degree of venous congestion and had good interpretability. However, the relatively small sample size (*n* = 80) limits its generalizability. Previous studies have found that decision tree models such as CART are prone to overfitting when the sample size is insufficient or the data distribution is single which can affect the robustness of the model (25, 27). Although we used the test set for internal validation, the lack of external data validation limits the applicability of the model in a wider range of clinical settings. In addition, the performance of the model may be affected by specific characteristics of the population in this study, such as the severity of the disease, sedation strategy, and ICU treatment practice. In the future, multicenter and large-sample studies are needed to verify the stability of this model and enhance its application value in different clinical settings.

Due to its interpretability and ability to capture interactions among variables, the CART algorithm has been widely applied in medical research in recent years. This study selected CART primarily for its advantage in visualizing decision pathways, which facilitates clinicians' understanding of the hierarchical relationships among variables. Our study found that when IVCdmax exceeded 1.975 cm, the model preferentially classified patients into a higher VExUS grade, a stratification logic that intuitively reflects the hemodynamic characteristics of venous congestion. With IVCdmax serving as the root node, its pivotal role is consistent with several studies (28, 29) demonstrating that IVC dilation is a direct sign of elevated RAP and is significantly associated with right ventricular dysfunction (e.g., reduced TAPSE, increased right atrial area). Although TAPSE and the tricuspid E-wave peak are of secondary importance, they play a key role in distinguishing between VExUS grades 2 and 3. ROC curve analysis revealed that the AUC was 0.917 for VExUS grade 0 and 0.828 for grade 1, while the AUC reached 1.0 for grades 2 and 3, indicating that the model has excellent discriminatory ability for moderate-to-severe venous congestion. This is consistent with previous studies (20, 30) that have established the physiological basis of VExUS grading, wherein abnormal hepatic and portal venous Doppler waveforms (grade 2 or above) are highly associated with RAP >12 mmHg and significantly linked to the risk of acute kidney injury.

Although research on the correlation between right ventricular function and VExUS grading in IMV patients has made progress, this study has several limitations: the sample size was relatively small, ventilation parameters such as transpulmonary pressure and driving pressure were not included, and the temporal relationship between changes in VExUS grading and right ventricular function was not dynamically tracked. Additionally, deep sedation was used to eliminate spontaneous breathing during ultrasound acquisition to reduce patient–ventilator interaction and stabilize intrathoracic pressure, improving measurement consistency. However, this sedation may have influenced venous tone by increasing venous compliance and lowering right ventricular preload, potentially affecting VExUS grading. Future studies with larger sample sizes are warranted to explore whether higher VExUS thresholds, such as grades ≥2, offer greater specificity in identifying clinically significant venous congestion and its impact on right ventricular function. Moreover, future research should further investigate the relationship between VExUS grading and RV–pulmonary arterial coupling and aim to develop the right ventricular protective ventilation strategies tailored to VExUS grading.

## 5 Conclusion

This study aimed to develop a right ventricular function assessment model for IMV patients based on VExUS grading and the CART algorithm, thereby providing clinicians with a reliable tool to better monitor and manage the right ventricular function in critically ill patients. The results demonstrated that VExUS grading can effectively reflect changes in the patient's circulatory status, while the CART algorithm offers an excellent visual model for the quantitative evaluation of right ventricular function. This model not only helps enhance the precision of clinical decision-making, but also provides new insights and methodologies for future related research.

## Data Availability

The original contributions presented in the study are included in the article/Supplementary Material, further inquiries can be directed to the corresponding author/s.
