# Supplementary material for: Construction of a right ventricular function assessment model in patients undergoing invasive mechanical ventilation based on VExUS grading and the classification and regression tree algorithm
Source: Front Cardiovasc Med. 2025 Sep 4;12:1608210. doi: 10.3389/fcvm.2025.1608210 (PMC12443755; doi:10.3389/fcvm.2025.1608210)
Supplement: Supplementary file 1 [file Image1.pdf]

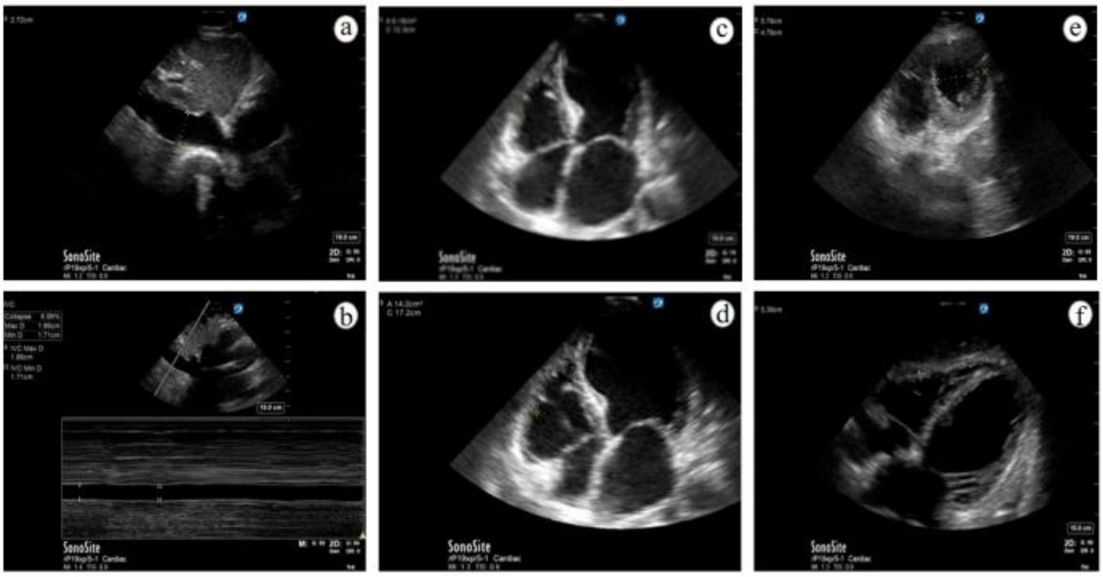

Supplementary Figure 1 Measurement of Echocardiographic Parameters for Right Heart Preload. Panels a-b show the inferior vena cava (IVC) diameter at end-expiration (minimum) and end-inspiration (maximum), used to calculate the IVC collapsibility index (△IVC) = (IVC end-inspiration – IVC end-expiration) / IVC end-expiration, with a normal value >18%. Panels c-d display the right ventricular end-diastolic area (RVEDA), right atrial end-diastolic area (RAEDA), right ventricular end-systolic area (RVESA), and left ventricular end-diastolic area (LVEDA). Panel e shows the eccentricity index (EI), calculated as the ratio of the long-axis diameter to the short-axis diameter, with a normal value of ≤1. Panel f illustrates the right ventricular free wall thickness (RVFW), with a normal value of ≤5 mm.
