# Supplementary material for: Construction of a right ventricular function assessment model in patients undergoing invasive mechanical ventilation based on VExUS grading and the classification and regression tree algorithm
Source: Front Cardiovasc Med. 2025 Sep 4;12:1608210. doi: 10.3389/fcvm.2025.1608210 (PMC12443755; doi:10.3389/fcvm.2025.1608210)
Supplement: Supplementary file 2 [file Image2.pdf]

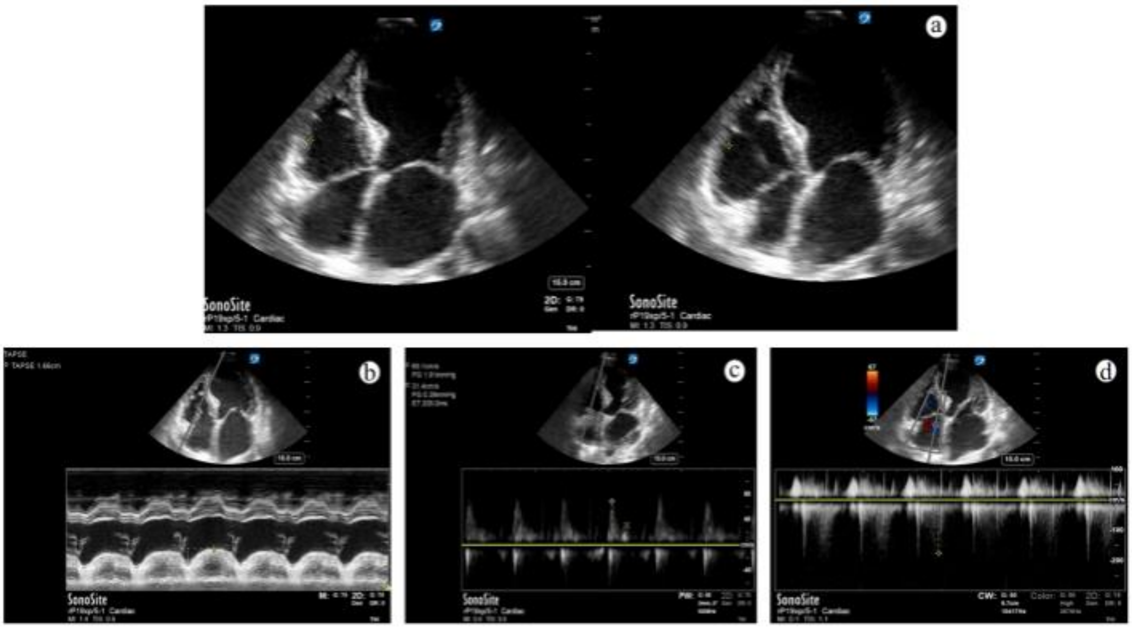

Supplementary Figure 2 Measurement of Echocardiographic Parameters for Right Ventricular Systolic and Diastolic Function and Afterload. Panel a shows the fractional area change (FAC), calculated as (RVEDA − RVESA) / RVEDA, with a normal value >40%. Panel b displays the tricuspid annular plane systolic excursion (TAPSE), with a normal value ≥17 mm. Panel c shows the early (E) and late (A) peak diastolic velocities across the tricuspid valve, used to calculate the E/A ratio. The reference value for E is 57±8 cm/s and for A is 39±6 cm/s, with a normal E/A ratio >1. Panel d presents the peak tricuspid regurgitant velocity (TRVmax), used to estimate pulmonary artery systolic pressure (PASP) using the formula PASP = $4 \times (TRVmax)^2$ + right atrial pressure (RAP), with a normal value <30 mmHg.
