# Supplementary material for: Construction of a right ventricular function assessment model in patients undergoing invasive mechanical ventilation based on VExUS grading and the classification and regression tree algorithm
Source: Front Cardiovasc Med. 2025 Sep 4;12:1608210. doi: 10.3389/fcvm.2025.1608210 (PMC12443755; doi:10.3389/fcvm.2025.1608210)
Supplement: Supplementary file 3 [file Image3.pdf]

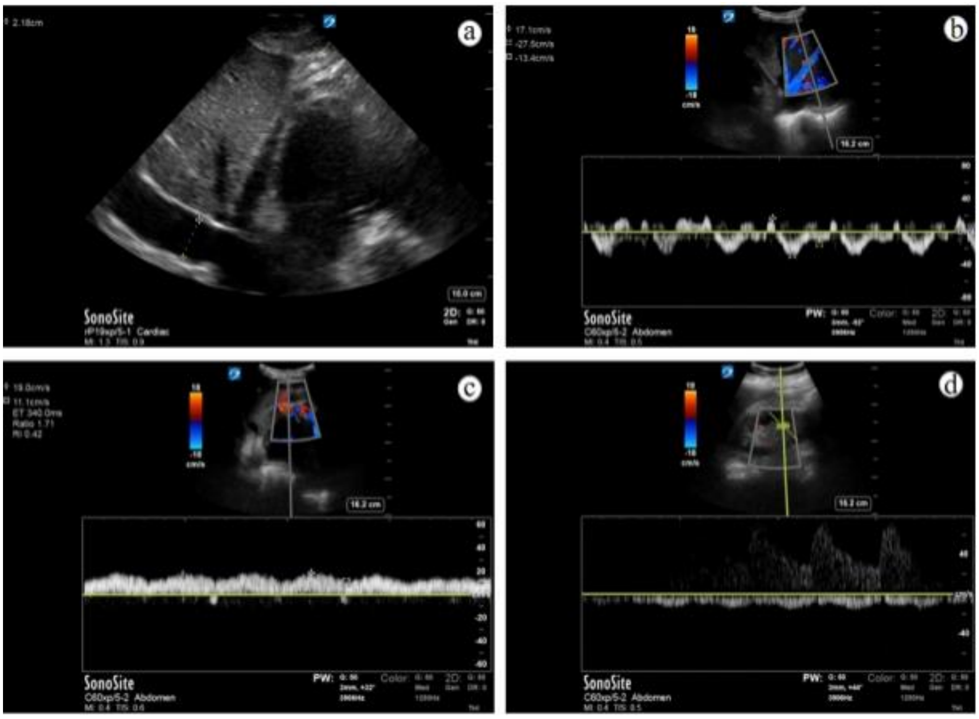

Supplementary Figure 3 Measurement of VExUS Grading Ultrasound Parameters. Panel a shows the maximum diameter of the inferior vena cava (IVCdmax). Panel b presents the hepatic vein Doppler waveform (preferably from the middle hepatic vein), recording the retrograde A wave and the antegrade S and D waves. When the amplitude of the S wave is greater than that of the D wave, the hepatic vein Doppler is considered normal. If the S wave amplitude is less than the D wave but remains below the baseline, it is considered mildly abnormal. If the S wave is reversed, it is classified as severely abnormal. Panel c shows the peak systolic (Vmax) and diastolic (Vmin) flow velocities in the main trunk of the portal vein. The pulsatility index is calculated as (Vmax − Vmin) / Vmax. A pulsatility index $<30\%$ is considered normal, 30%–50% is mildly abnormal, and $\geq 50\%$ is classified as severely abnormal. Panel d displays the intrarenal vein Doppler signal (preferably from the right kidney). A continuous, low-pulsatility waveform without interruption is considered normal. A pulsatile waveform with distinct S and D waves is considered mildly abnormal, while a monophasic waveform with only a D wave is classified as severely abnormal.
