# Supplementary material for: Construction of a right ventricular function assessment model in patients undergoing invasive mechanical ventilation based on VExUS grading and the classification and regression tree algorithm
Source: Front Cardiovasc Med. 2025 Sep 4;12:1608210. doi: 10.3389/fcvm.2025.1608210 (PMC12443755; doi:10.3389/fcvm.2025.1608210)
Supplement: Supplementary file 4 [file Image4.pdf]

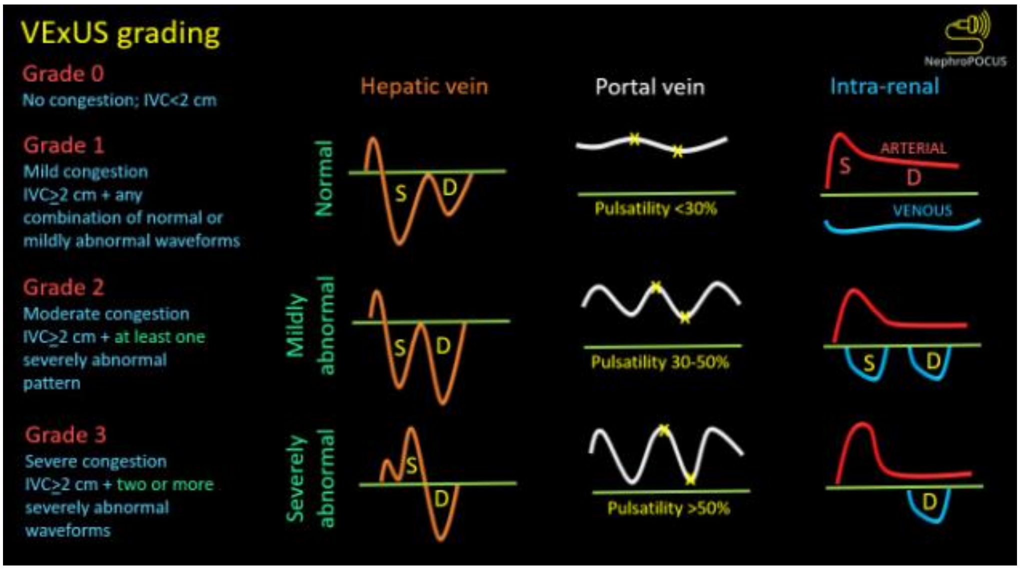

Supplementary Figure 4 Illustration of VExUS Grading. In the VExUS grading system, when the maximum diameter of the inferior vena cava (IVCdmax) is less than 2 cm, VExUS is classified as grade 0, indicating no systemic congestion. When IVCdmax exceeds 2 cm, the degree of congestion is further classified into VExUS grades 1 to 3 based on the severity of Doppler abnormalities in the hepatic, portal, and intrarenal veins [1,2].

[1] Koratala A, Ibrahim M, Gudlawar S. VExUS to Guide Ultrafiltration in Hemodialysis: Exploring a Novel Dimension of Point of Care Ultrasound [J]. Pocus j, 2024, 9(1): 16-19.

[2] Koratala A, Romero-González G, Soliman-Aboumarie H, *et al*. Unlocking the Potential of VExUS in Assessing Venous Congestion: The Art of Doing It Right [J]. Cardiorenal Med, 2024, 14(1): 350-374.
